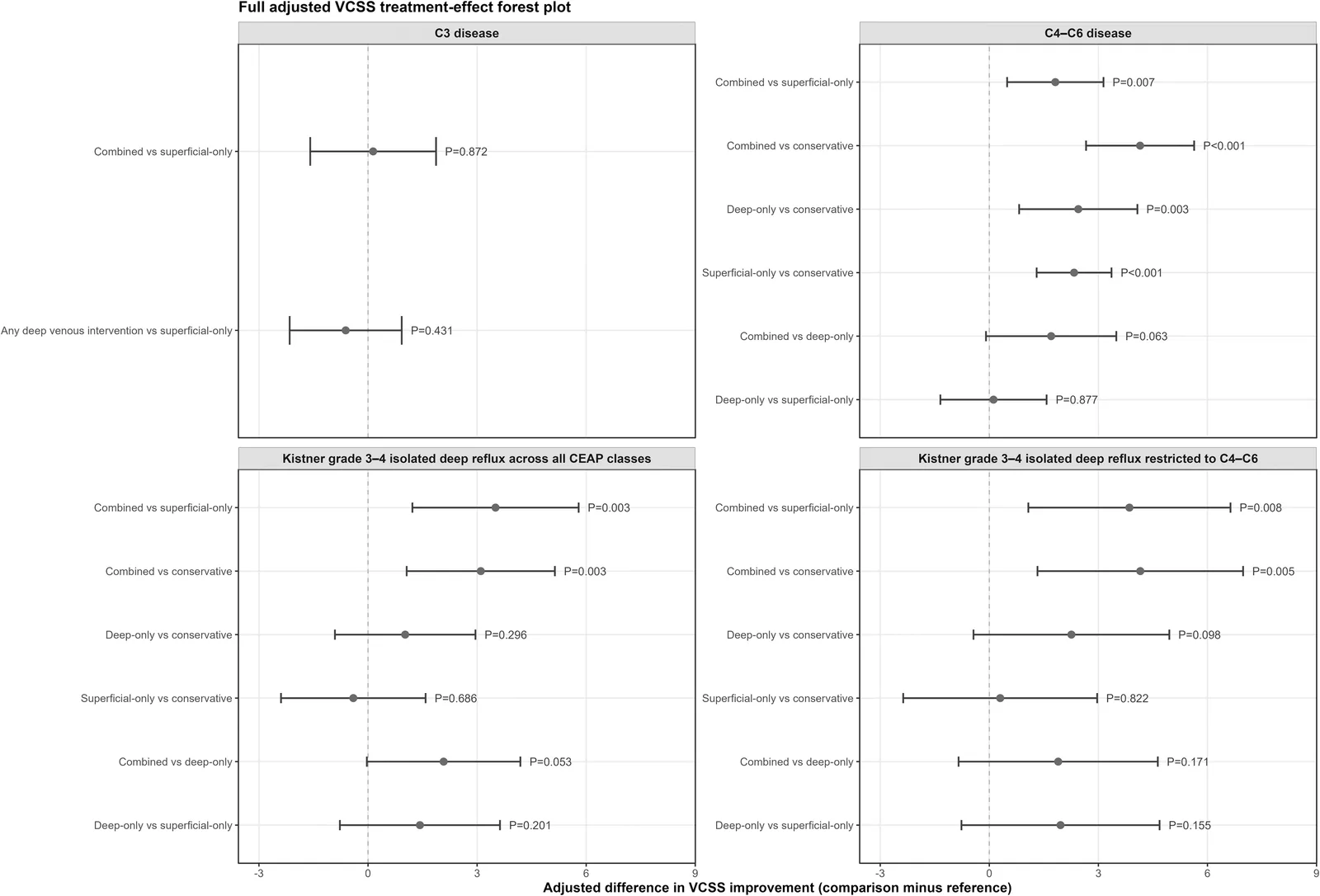# CEAP-guided and Kistner-refined selection of deep venous intervention for primary lower-limb venous valvular incompetence: A two-center cohort study

**DOI:** 10.1016/j.jvsv.2026.102604

**Published:** 2026-08-24

**Authors:** Baiqiang Zhang, Jinghe Zhu, Sijing Wang, Renjie Bai, Yang Li, Hui Zhao, Jie Zhang, Liang Zhao, Menglong Li, Yingfeng Wu

**Affiliations:** a Department of Vascular Surgery, Xuanwu Hospital, Capital Medical University, Beijing, P.R. China; b Department of Vascular Surgery, Beijing Luhe Hospital, Capital Medical University, Beijing, P.R. China; c Evidence-based Medicine Centre, Beijing Luhe Hospital, Capital Medical University, Beijing, P.R. China

**Keywords:** Chronic venous disease, Deep venous reflux, Superficial venous reflux, Kistner grade, Venous Clinical Severity Score, Treatment strategy

## Abstract

**Objective:**

To evaluate whether the Clinical-Etiological-Anatomical-Pathophysiological (CEAP) clinical class and Kistner grade could guide the selection of deep venous intervention for primary lower-limb venous valvular incompetence.

**Methods:**

A two-center retrospective cohort study of 578 consecutive eligible limbs with primary lower-limb venous valvular incompetence and available venographic and clinical records between 2015 and 2025 categorized into the following four groups was conducted: conservative management (n = 241), superficial-only treatment (n = 240), deep-only treatment (n = 44), and combined treatment (n = 53). Deep intervention mainly consisted of femoral vein valvuloplasty; combined treatment comprised femoral valvuloplasty plus superficial treatment, most often radiofrequency ablation, with high ligation and stripping performed when indicated. The primary outcome was Venous Clinical Severity Score (VCSS) improvement. Analyses were conducted across several populations: hierarchically in the C3 group, the overall C4-C6 population, the C4-C6 Kistner grade 3-4 isolated deep reflux subgroup, and an all-CEAP Kistner grade 3-4 sensitivity population.

**Results:**

In the C3 group, combined treatment provided no additional VCSS benefit compared with superficial venous-only treatment (adjusted β, .1; 95% CI, −1.6 to 1.9; *P* = .872). However, in the C4-C6 population, combined treatment achieved greater VCSS improvement than superficial venous-only treatment (adjusted β, 1.8; 95% CI, 0.5-3.1; *P* = .007). In C4-C6 limbs with Kistner grade 3-4 isolated deep venous reflux, combined treatment showed greater benefit than both superficial venous-only treatment (adjusted β, 3.9; 95% CI, 1.1-6.6; *P* = .008) and conservative management (adjusted β, 4.2; 95% CI, 1.3-7.0; *P* = .005). All CEAP Kistner grade 3-4 sensitivity analyses showed directionally consistent results.

**Conclusions:**

Selective femoral vein valvuloplasty combined with superficial venous treatment may be clinically justified in C4-C6 disease, especially with Kistner grades 3-4 reflux; however, it is not supported in C3 disease.

**Article Highlights**

• **Type of Research:** Two-center retrospective cohort study
• **Key Findings:** Among 578 limbs, combined superficial and deep venous intervention showed no additional Venous Clinical Severity Score improvement over superficial-only intervention in patients with C3 disease (*P* = .872). However, combined intervention showed greater improvement in C4-C6 disease (adjusted β, 1.82; *P* = .007), particularly in cases of isolated deep reflux with Kistner grade 3-4 (β 3.85; *P* = .008).
• **Take Home Message:** Clinical-Etiological-Anatomical-Pathophysiological clinical class may serve as the primary framework for selecting deep venous intervention, with Kistner grade as a supplementary refinement rather than a stand-alone indication; prospective validation is required.

Chronic venous disease (CVD) is a common lower-limb vascular disorder ranging from varicose veins and edema to skin changes and venous ulceration.^1^^,^^2^ Its pathophysiology reflects the combined effects of valvular incompetence, venous reflux, stasis, and sustained venous hypertension, leading to impaired quality of life and substantial health care expenditure.^1^, ^2^, ^3^, ^4^, ^5^, ^6^, ^7^ Chronic venous insufficiency affects approximately 2% to 7% of adults, venous ulceration affects 0.5% to 1.5%, and ulcer recurrence may reach 54% to 78%.^1^^,^^2^^,^^8^ The economic burden is particularly substantial for venous leg ulceration, the most advanced and resource-intensive end of CVD.

Clinical stratification is central to treatment decision-making. The Clinical-Etiological-Anatomical-Pathophysiological (CEAP) classification describes clinical manifestations across clinical classes C0-C6, whereas the Venous Clinical Severity Score (VCSS) quantifies symptom and sign burden.^9^^,^^10^ However, clinical severity and imaging-defined reflux are not interchangeable.^11^^,^^12^ Substantial reflux may be present on imaging despite limited clinical burden, and venography and ultrasound study may classify reflux phenotypes differently.^11^^,^^12^ Treatment escalation based only on imaging findings may therefore overestimate expected benefit.

Superficial venous surgery is usually prioritized in patients with combined deep and superficial venous reflux, whereas the role of deep venous intervention, particularly femoral vein valve repair for primary deep valvular incompetence, remains less clearly defined.^13^ Current guidelines still generally favor superficial venous surgery first and remain cautious about deep venous valve repair.^14^ However, this strategy does not clearly define whether selected patients with higher CEAP clinical classes may benefit from combined deep and superficial intervention rather than superficial venous surgery alone. Moreover, much of the evidence supporting deep venous reconstruction is derived from relatively old and small studies.^13^^,^^14^ Therefore, an important clinical question remains whether CEAP clinical class can help identify patients in whom combined treatment provides additional clinical benefit.

Accordingly, we evaluated whether combined deep and superficial intervention was associated with greater VCSS improvement than superficial-only intervention in patients with C4-C6 primary lower-limb venous valvular incompetence, and whether venography-defined isolated deep reflux of Kistner grade 3-4 further identified a subgroup with greater benefit. In the Kistner descending venographic classification, grade 3 reflux extends below the knee into the calf, whereas grade 4 reflux reaches the ankle.^15^

## Methods

### Study design and population

This two-center retrospective cohort study included 578 consecutive eligible limbs with primary lower-limb venous valvular incompetence and available clinical records from August 2015 to November 2025. All included limbs underwent both ascending and descending venography. “Consecutive” refers to all eligible limbs within this predefined venography-based sampling frame rather than all patients with CVD treated at the two centers. In routine clinical practice, duplex ultrasound study was the first-line imaging examination, whereas venography was reserved for patients requiring additional anatomical or hemodynamic assessment, as detailed below.

Patients with post-thrombotic syndrome, clinically significant iliac venous obstruction or compression, pelvic congestion syndrome, ovarian or spermatic varices, nutcracker syndrome, or symptomatic lower-limb atherosclerotic occlusive disease were excluded. The exclusion of post-thrombotic, iliocaval obstructive or compressive, and pelvic venous disorders was based on clinical history, iliac venous duplex ultrasound study, and bilateral lower-extremity and iliocaval venography.

Follow-up duration was calculated from the date of surgery for surgically treated limbs or from the initiation of the standardized conservative treatment protocol for conservatively managed limbs to the last available VCSS assessment between February and March 2026. All 578 limbs were included in venography-based analyses; ultrasound-related and concordance analyses included 569 limbs after exclusion of nine limbs without usable ultrasound data.

The CEAP clinical class was used as the primary stratification variable. Limbs were grouped as C0-C2 (n = 90), C3 (n = 127), and C4-C6 (n = 361). CEAP-stratified treatment-effect analyses focused on the C3 and C4-C6 populations. The main Kistner-refined analysis was restricted to C4-C6 limbs with venography-defined isolated deep reflux of Kistner grade 3-4, and the corresponding all-CEAP population was examined as a sensitivity analysis.

The protocol of this study was approved by the Institutional Review Board of Beijing Luhe Hospital, Capital Medical University (No. 2024-LHKY-068-02), and conducted in accordance with the Declaration of Helsinki.^16^ The requirement for written informed consent was waived by the Institutional Review Board because of the retrospective study design and the use of de-identified, routinely collected clinical data.

### Imaging assessment and reflux phenotype definitions

At both centers, venous duplex ultrasound study and venography were performed according to the same institutional standards. Duplex ultrasound study was the first-line examination for deep and superficial venous reflux, and examinations and formal reports were completed by specialist physicians experienced in vascular ultrasound study. Reflux was assessed using pulsed-wave spectral Doppler according to both duration and anatomical location. Retrograde flow lasting >1.0 second in the common femoral, femoral, or popliteal vein and >0.5 second in the profunda femoris or calf deep veins was considered pathologic reflux.^12^^,^^14^ Assessment also included venous patency and, when the leaflets were adequately visualized, valve morphology and function, including leaflet motion, coaptation, and flow direction. Findings suggestive of previous thrombosis, luminal narrowing, or venous outflow obstruction were recorded.

When patients could stand safely, reflux was assessed in the standing position with the examined limb relaxed and predominantly non-weight-bearing; a seated or reverse Trendelenburg position was used otherwise. Reflux in the common femoral vein, saphenofemoral junction, and proximal femoral vein was provoked using the Valsalva maneuver or distal limb augmentation. Reflux in the mid and distal femoral, popliteal, and calf deep veins was provoked by distal manual calf compression and rapid release or pneumatic cuff inflation and rapid deflation.^12^^,^^14^ No fixed time-of-day protocol was used.

For descriptive analyses, reflux duration was categorized using an investigator-defined scale: grade 0, no reflux; grade 1, >0 to 1 second; grade 2, >1 to 2 seconds; grade 3, >2 to 3 seconds; grade 4, >3 to 5 seconds; and grade 5, >5 seconds. This scale was used solely for descriptive analyses and was not considered equivalent to the venography-based Kistner classification. Ultrasound classifications were derived from the available images and contemporaneous formal reports. Primary valvular reflux was defined as pathologic reflux in a patent vein without sonographic evidence of previous thrombosis. Limbs showing venous wall thickening, residual thrombotic material, intraluminal webs or synechiae, recanalization, or luminal irregularity or narrowing were considered post-thrombotic and were excluded.

All 578 included limbs underwent both ascending and descending venography. In routine clinical practice, venography was selectively undertaken in patients requiring additional anatomical or hemodynamic assessment because of suspected deep venous valvular incompetence, complex or discordant clinical and duplex findings, consideration of deep venous reconstruction, or the need to exclude post-thrombotic, iliocaval obstructive or compressive, and pelvic venous disorders. Ascending venography was performed with the patient supine and covered the bilateral lower-extremity venous systems, iliac veins, and inferior vena cava; approximately 150 to 200 mL of nonionic contrast medium was administered during the complete venographic examination. Descending venography was used to assess deep venous valve morphology and the distal extent of reflux according to the Kistner method, with the patient at a 60° semiupright angle.^15^ A 5F catheter was introduced through the ipsilateral common femoral vein and positioned immediately proximal to the highest femoral valve and distal to the profunda femoris confluence. During a sustained Valsalva maneuver generating a pressure of >40 cm H_2_O, 20 to 25 mL of nonionic contrast medium was injected at 4 to 5 mL/s, and digital subtraction angiography was used to trace the distal extent of reflux. Kistner grade 0 indicated no significant reflux below the common femoral vein; grade 1 denoted reflux confined to the upper thigh; grade 2 indicated reflux extending into the lower thigh but remaining above the knee; grade 3 indicated reflux extending below the knee into the calf; and grade 4 represented reflux extending through the calf toward the ankle. Grades 3-4 were considered higher-grade deep venous reflux for imaging-stratified analyses.

Venographic images and contemporaneous reports were independently reviewed by two senior vascular physicians blinded to patient identity and patient-specific clinical information. Disagreements were adjudicated by a third physician. A fourth physician prepared the imaging materials but did not participate in their interpretation or final classification.

Reflux phenotypes were classified as no definite reflux, isolated deep reflux, isolated superficial reflux, or combined deep and superficial reflux, depending on the venous systems involved. Ultrasound-derived phenotypes were retrospectively compared with venographic classifications in limbs with usable paired data. Cross-modality concordance was defined as exact agreement between the two modalities in the assigned reflux phenotype. Complete cross-modality discordance was defined as isolated deep reflux on one modality and isolated superficial reflux on the other.

### Treatment strategies and outcomes

Limbs were classified as receiving conservative management, superficial venous-only treatment, deep venous-only treatment, or combined deep and superficial treatment. Conservative management was defined as nonoperative treatment without superficial or deep venous intervention. For these cases, the index date was the date on which nonoperative management was selected and the standardized protocol was initiated. At both centers, this protocol comprised compression therapy, venoactive medication, avoidance of prolonged sitting or standing, and regular walking exercise. Thigh-high compression stockings were generally recommended, with compression bandaging used when stockings were impractical, particularly in patients with active venous ulceration. Adherence was assessed during routine follow-up and was generally good, although it was not quantified using a formal scale.

Treatment allocation was nonrandomized and not governed by a prespecified escalation protocol. Shared decisions incorporated clinical manifestations, reflux distribution and severity, venous anatomy, reconstructive feasibility, operative risk, previous treatment, surgeon judgment, and patient preference.

The superficial venous procedure was selected according to saphenous anatomy and technical suitability. Radiofrequency ablation was generally preferred for a sufficiently straight target vein suitable for safe catheter-based treatment. High ligation and stripping were used when endovenous thermal ablation was unsuitable or potentially unsafe because of marked tortuosity, a very superficial course, focal aneurysmal dilatation, marked venous dilatation, or a very large caliber combined with other unfavorable anatomical features.^13^^,^^17^ No single diameter threshold was used. Adjunctive phlebectomy, sclerotherapy, or perforator treatment was performed as indicated.

Deep venous surgery was undertaken for primary deep venous valvular incompetence. Femoral vein internal valvuloplasty was the predominant technique; valve transplantation or other reconstruction was used when the native valve was absent, severely damaged, or unsuitable.^18^ Under general anesthesia, the common femoral, profunda femoris, and femoral veins were exposed through an approximately 10-cm upper-medial-thigh incision. After systemic heparinization and proximal and distal vascular control, a longitudinal venotomy was made over the first accessible incompetent femoral valve. Elongated or prolapsed leaflets were shortened and suspended with 6-0 polypropylene sutures to restore coaptation, and the venotomy was closed. Patency and valve competence were assessed using the intraoperative strip test (Kistner test). The femoral vein distal to the repaired valve was temporarily occluded, and the blood column was milked proximally across the valve segment. After release of the stripping hand, immediate retrograde refilling with distal venous distension indicated residual reflux, whereas unobstructed flow without retrograde refilling indicated satisfactory valve competence.^19^ External sleeve wrapping was used selectively when venous wall dilatation or inadequate wall support was considered likely to compromise restored valve coaptation. A 1-cm-wide polyester-urethane vascular patch was placed around the repaired segment without causing luminal stenosis and was sutured with slight laxity rather than under tension to allow limited venous expansion (Supplementary Fig 1, online only).^18^

Postoperative adjunctive treatment was standardized at both centers for approximately 3 to 6 months and included thigh-high compression, bandaging when stockings were impractical, venoactive medication unless contraindicated, avoidance of prolonged sitting or standing, and regular walking exercise. Adherence was assessed during routine follow-up and was generally good, although it was not formally quantified using a specific scale.

The primary outcome was VCSS improvement, calculated as the baseline VCSS minus the last available follow-up VCSS; higher positive values indicated greater clinical improvement.

### Statistical analysis

Before analysis, prespecified missing or indeterminate codes were recoded as missing. Continuous variables are presented as mean ± standard deviation or median with interquartile range, and categorical variables as frequency and percentage. Baseline characteristics were compared using Kruskal-Wallis tests for continuous variables and *χ*^2^ tests for categorical variables. Associations between CEAP clinical class, baseline VCSS, and imaging-derived reflux grades were assessed using Spearman rank correlation.

Because treatment was not randomized, primary inference was based on CEAP-stratified multivariable models rather than unadjusted comparisons.^20^ Multivariable linear regression was used with VCSS improvement as the dependent variable and treatment group as the primary independent variable. Models were adjusted for baseline VCSS, follow-up duration, and study center, representing the two participating hospitals.

In the C3 population, both a four-group model and a secondary three-group model were used. The four-group model included conservative management, superficial-only intervention, deep-only intervention, and combined deep and superficial intervention. In the three-group model, the deep-only and combined intervention groups were merged as any deep venous intervention. The C4-C6 and Kistner grade 3-4 analyses retained all four treatment groups.

CEAP clinical class was not included in the CEAP-stratified models but was included as an additional covariate in the all-CEAP Kistner grade 3-4 sensitivity model. In the four-group models, prespecified pairwise contrasts were estimated from the same fitted model. Positive adjusted coefficients indicated greater VCSS improvement in the first-listed comparison group than in the reference group. Adjusted coefficients, 95% confidence intervals, and two-tailed *P* values were reported. Treatment effects therefore represent adjusted between-group differences in cumulative VCSS improvement over observed follow-up, rather than time-standardized rates or longitudinal time-varying effects.^21^

Complete-case analysis was applied according to the variables required for each model; consequently, model sample sizes varied according to data availability within each prespecified analysis population. Supplementary analyses included full adjusted treatment comparisons and imaging-concordance analyses. Venography-ultrasound reflux phenotype concordance was assessed among limbs with paired usable imaging data using exact agreement and Cohen's kappa. The frequency of complete deep vs superficial discordance was also reported descriptively. All analyses and graphics were performed using R software, version 4.4.1 (R Foundation for Statistical Computing). A two-tailed *P* value <.05 was considered statistically significant.

## Results

### Study cohort and baseline characteristics

The cohort included 578 limbs: 241 managed conservatively, 240 treated with superficial-only intervention, 44 with deep-only intervention, and 53 with combined intervention (Fig 1). CEAP classes C0-C2, C3, and C4-C6 accounted for 90, 127, and 361 limbs, respectively. Baseline characteristics are summarized in Table I. Age, center, CEAP clinical class, and baseline VCSS differed across treatment groups. The mean baseline VCSS was 6.5 ± 4.6 overall, ranging from 3.9 ± 3.6 in the conservative group to 9.2 ± 5.1 in the deep-only group. The median follow-up duration was 33.7 months and was similar across groups.

**Fig 1:**
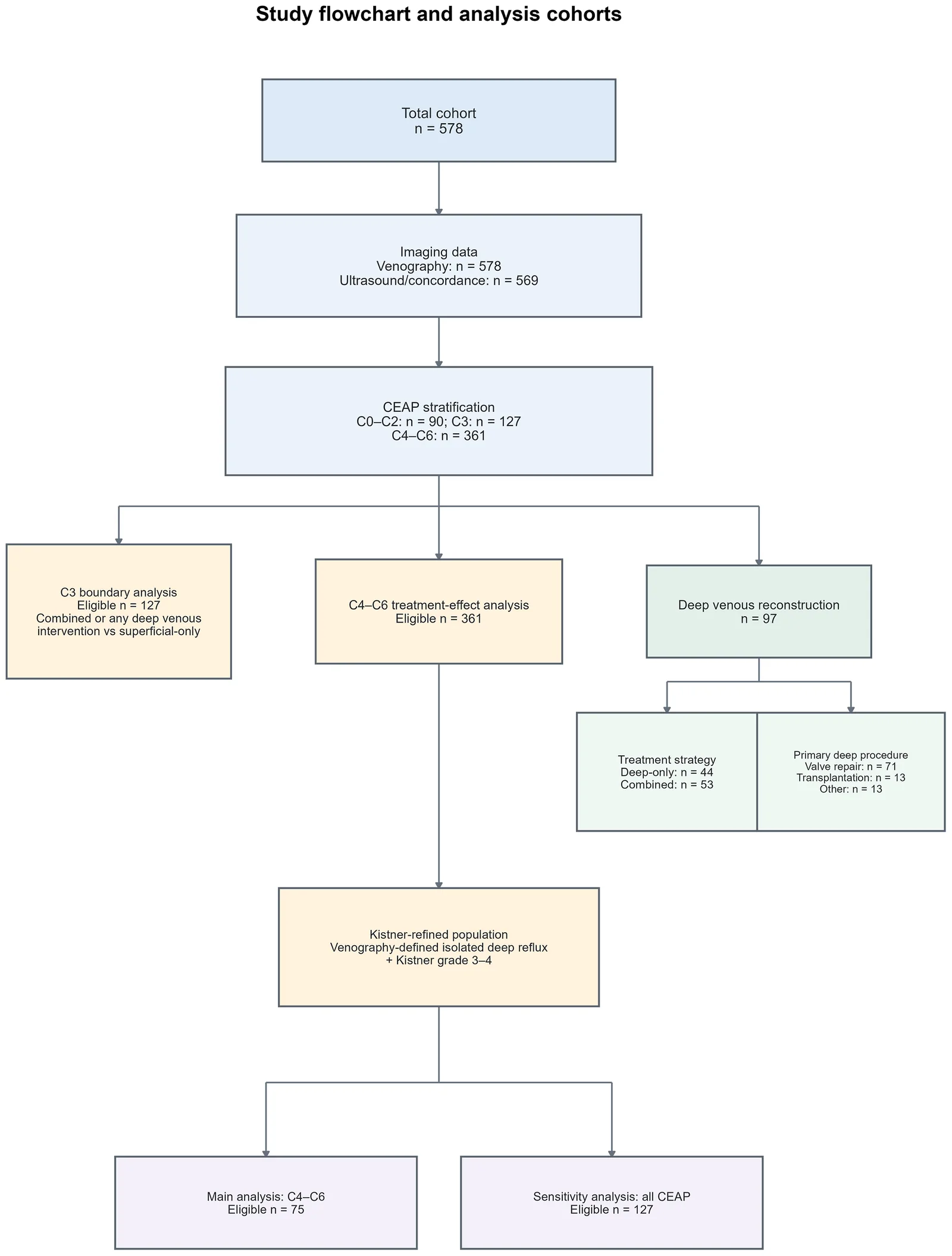
Study flowchart and analysis cohorts. The flowchart summarizes the overall cohort, available venographic and ultrasound data, CEAP clinical-class strata, principal CEAP-stratified analyses, the deep venous reconstruction cohort, and the Kistner-refined analysis populations. The 97 limbs undergoing deep venous reconstruction are classified by treatment strategy (deep-only intervention or combined deep and superficial intervention) and by primary deep venous procedure (valve repair, valve transplantation, or other reconstruction). The Kistner-refined population comprised limbs with venography-defined isolated deep reflux of Kistner grade 3-4; the C4-C6 population was used for the main analysis, and the corresponding all-CEAP population was used for sensitivity analysis. All counts refer to limbs. Adjusted analyses used complete cases for the variables required in each model; therefore, sample sizes may differ across analyses. *CEAP*, Clinical-Etiological-Anatomical-Pathophysiological.

**Table I:** Baseline characteristics of the study cohort by treatment group

Characteristic	Category	Overall	Conservative	Deep-only	Superficial-only	Combined deep and superficial	*P* value
No. of limbs		578	241	44	240	53	
Age, years		61.89 ± 11.55	61.72 ± 12.11	65.77 ± 9.50	62.32 ± 10.32	57.51 ± 14.28	.044
Sex	Male	374 (64.7)	157 (65.1)	36 (81.8)	148 (61.7)	33 (62.3)	.08
	Female	204 (35.3)	84 (34.9)	8 (18.2)	92 (38.3)	20 (37.7)	
Limb side	Left	293 (50.7)	111 (46.1)	25 (56.8)	128 (53.3)	29 (54.7)	.29
	Right	285 (49.3)	130 (53.9)	19 (43.2)	112 (46.7)	24 (45.3)	
Body mass index, kg/m^2^		27.02 ± 3.97	26.65 ± 3.81	26.98 ± 4.02	27.49 ± 4.07	26.69 ± 4.05	.348
Hypertension	Yes	257 (46.1)	105 (45.3)	20 (45.5)	111 (48.5)	21 (39.6)	.685
	No	301 (53.9)	127 (54.7)	24 (54.5)	118 (51.5)	32 (60.4)	
Diabetes mellitus	Yes	119 (21.3)	50 (21.6)	11 (25.0)	45 (19.7)	13 (24.5)	.785
	No	439 (78.7)	182 (78.4)	33 (75.0)	184 (80.3)	40 (75.5)	
Center	Center 1	443 (76.6)	181 (75.1)	29 (65.9)	196 (81.7)	37 (69.8)	.048
	Center 2	135 (23.4)	60 (24.9)	15 (34.1)	44 (18.3)	16 (30.2)	
CEAP clinical class	C0	36 (6.2)	35 (14.5)	0 (0.0)	1 (0.4)	0 (0.0)	<.001
	C1	6 (1.0)	4 (1.7)	0 (0.0)	2 (0.8)	0 (0.0)	
	C2	48 (8.3)	30 (12.4)	1 (2.3)	17 (7.1)	0 (0.0)	
	C3	127 (22.0)	68 (28.2)	10 (22.7)	34 (14.2)	15 (28.3)	
	C4	222 (38.4)	77 (32.0)	15 (34.1)	112 (46.7)	18 (34.0)	
	C5	39 (6.7)	15 (6.2)	1 (2.3)	20 (8.3)	3 (5.7)	
	C6	100 (17.3)	12 (5.0)	17 (38.6)	54 (22.5)	17 (32.1)	
Baseline VCSS		6.46 ± 4.64	3.86 ± 3.57	9.20 ± 5.09	8.09 ± 4.43	8.60 ± 3.78	<.001
Follow-up duration, months		33.65 [20.12, 51.71]	32.61 [16.32, 51.57]	36.18 [19.15, 65.90]	33.46 [17.30, 50.72]	35.84 [26.32, 51.61]	.36

*CEAP*, Clinical-Etiological-Anatomical-Pathophysiological; *IQR*, interquartile range; *SD*, standard deviation; *VCSS*, Venous Clinical Severity Score.

Data are presented as mean ± standard deviation, median [interquartile range], or n (%), as appropriate. All analyses were performed at the limb level. Percentages were calculated using nonmissing observations. Hypertension and diabetes mellitus data were available for 558 limbs. *P* values were calculated using the Kruskal-Wallis test for continuous variables and the *χ*^2^ test for categorical variables.

### Clinical severity, reflux phenotype, and imaging concordance

CEAP clinical class was strongly associated with baseline VCSS (Spearman ρ = 0.705; *P* < .001; Fig 2). By contrast, correlations between CEAP clinical class and imaging-defined reflux grades were weak or nonsignificant, including ultrasound deep reflux grade (ρ = 0.078; *P* = .063). Among 569 limbs with analyzable venography and ultrasound data, complete deep vs superficial discordance occurred in only 16 limbs (2.8%). Exact agreement across all four reflux phenotype categories was 57.6%, with Cohen's κ = 0.351. Most nonmatching classifications involved combined reflux on one modality and an isolated deep or superficial component on the other (Fig 2; Table II). Across the C0-C2, C3, and C4-C6 groups, the proportion of combined deep and superficial reflux increased progressively on both venography (33.3%, 41.7%, and 51.5%, respectively) and ultrasound study (30.3%, 39.7%, and 44.4%, respectively) (Fig 3, *A* and *B*; Table II). When either imaging modality was considered, combined deep and superficial reflux was present in 232 of 361 C4-C6 limbs (64.3%), indicating that coexistence of deep and superficial reflux was common in advanced clinical classes. Treatment distributions also differed across CEAP clinical classes: conservative management decreased from 76.7% in C0-C2 to 53.5% in C3 and 28.8% in C4-C6, whereas superficial-only intervention increased from 22.2% to 26.8% and 51.5%, respectively (Fig 3, *C*; Table II). Additional venographic reflux phenotype and Kistner grade distributions are provided in Supplementary Table I (online only).

**Fig 2:**
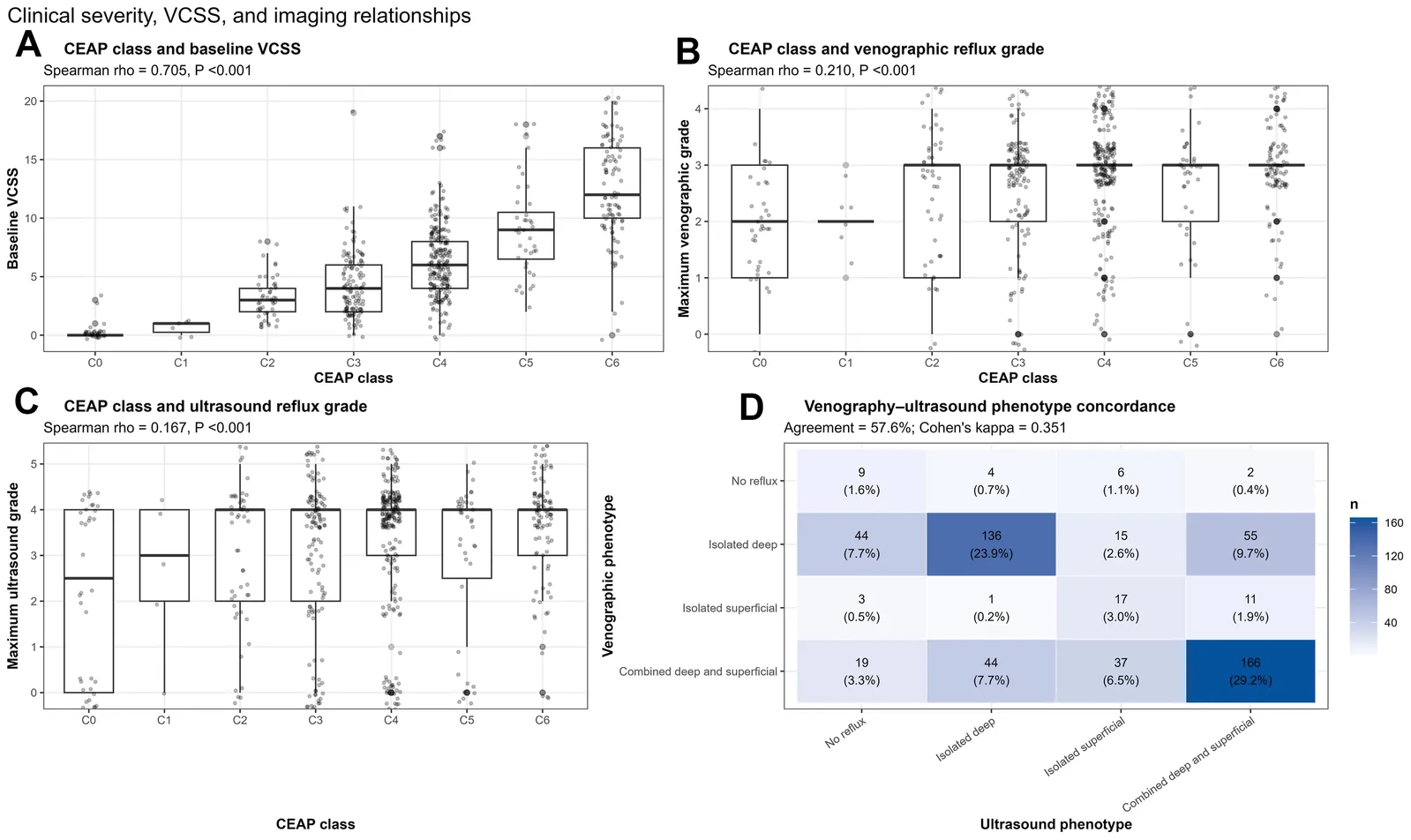
Clinical severity, VCSS, and imaging relationships. (A) Distribution of baseline VCSS across CEAP clinical classes. **B,** Distribution of the maximum venography-derived reflux grade across CEAP clinical classes. **C,** Distribution of the maximum ultrasound-derived reflux-duration grade across CEAP clinical classes. **D,** Cross-tabulation of reflux phenotypes classified by venography and duplex ultrasound study among limbs with usable paired imaging data. In panels **(A-C)** individual points represent limbs, *boxes* represent the interquartile range, and *horizontal lines* within the boxes represent medians. Spearman correlation coefficients and corresponding *P* values are shown above each panel. The ultrasound reflux-duration grading scale was investigator-defined and used only for descriptive analyses; it was not considered equivalent to the venography-based Kistner classification. In panel **(D)** rows represent venography-defined reflux phenotypes and columns represent ultrasound-defined reflux phenotypes. Each cell shows the number and percentage of limbs assigned to the corresponding combination of classifications. Percentages were calculated using the 569 limbs with usable paired ultrasound and venographic data as the denominator. Diagonal cells represent exact phenotype agreement, and color intensity reflects the number of limbs in each cell. *CEAP*, Clinical-Etiological-Anatomical-Pathophysiological; *VCSS*, Venous Clinical Severity Score.

**Table II:** Treatment strategies and imaging characteristics by CEAP clinical class

Characteristic	Category	Overall	C0-C2	C3	C4-C6
CEAP clinical-class strata	C0-C2	90 (15.6)			
	C3	127 (22.0)			
	C4-C6	361 (62.5)			
Treatment strategy	Conservative	241 (41.7)	69 (76.7)	68 (53.5)	104 (28.8)
	Deep-only	44 (7.6)	1 (1.1)	10 (7.9)	33 (9.1)
	Superficial-only	240 (41.5)	20 (22.2)	34 (26.8)	186 (51.5)
	Combined deep and superficial	53 (9.2)	0 (0.0)	15 (11.8)	38 (10.5)
Venographic reflux pattern	No definite reflux	21 (3.6)	3 (3.3)	7 (5.5)	11 (3.0)
	Isolated deep reflux	256 (44.3)	56 (62.2)	64 (50.4)	136 (37.7)
	Isolated superficial reflux	32 (5.5)	1 (1.1)	3 (2.4)	28 (7.8)
	Combined deep and superficial reflux	269 (46.5)	30 (33.3)	53 (41.7)	186 (51.5)
Ultrasound reflux pattern	No definite reflux	75 (13.2)	19 (21.3)	19 (15.1)	37 (10.5)
	Isolated deep reflux	185 (32.5)	34 (38.2)	45 (35.7)	106 (29.9)
	Isolated superficial reflux	75 (13.2)	9 (10.1)	12 (9.5)	54 (15.3)
	Combined deep and superficial reflux	234 (41.1)	27 (30.3)	50 (39.7)	157 (44.4)
Reflux pattern by any imaging modality	No definite reflux	9 (1.6)	1 (1.1)	3 (2.4)	5 (1.4)
	Isolated deep reflux	190 (32.9)	41 (45.6)	46 (36.2)	103 (28.5)
	Isolated superficial reflux	26 (4.5)	2 (2.2)	3 (2.4)	21 (5.8)
	Combined deep and superficial reflux	353 (61.1)	46 (51.1)	75 (59.1)	232 (64.3)
Venography-ultrasound concordance	Concordant reflux pattern between venography and ultrasound	328 (57.6)	40 (44.9)	73 (57.9)	215 (60.7)
	Discordant reflux pattern between venography and ultrasound	241 (42.4)	49 (55.1)	53 (42.1)	139 (39.3)
Complete cross-modality discordance	Present	16 (2.8)	3 (3.4)	6 (4.8)	7 (2.0)
	Absent	553 (97.2)	86 (96.6)	120 (95.2)	347 (98.0)
Venographic deep reflux/Kistner grade	Grade 0/no deep reflux	53 (9.2)	4 (4.4)	10 (7.9)	39 (10.8)
	Kistner grade 1	93 (16.1)	25 (27.8)	21 (16.5)	47 (13.0)
	Kistner grade 2	154 (26.6)	34 (37.8)	35 (27.6)	85 (23.5)
	Kistner grade 3	255 (44.1)	27 (30.0)	54 (42.5)	174 (48.2)
	Kistner grade 4	23 (4.0)	0 (0.0)	7 (5.5)	16 (4.4)
Ultrasound deep reflux grade	Grade 0/no reflux	150 (26.4)	28 (31.5)	31 (24.6)	91 (25.7)
	Grade 1	14 (2.5)	3 (3.4)	4 (3.2)	7 (2.0)
	Grade 2	87 (15.3)	21 (23.6)	25 (19.8)	41 (11.6)
	Grade 3	62 (10.9)	5 (5.6)	14 (11.1)	43 (12.1)
	Grade 4	217 (38.1)	28 (31.5)	39 (31.0)	150 (42.4)
	Grade 5	39 (6.9)	4 (4.5)	13 (10.3)	22 (6.2)
Ultrasound superficial reflux grade	Grade 0/no reflux	260 (45.7)	53 (59.6)	64 (50.8)	143 (40.4)
	Grade 1	5 (0.9)	0 (0.0)	1 (0.8)	4 (1.1)
	Grade 2	44 (7.7)	7 (7.9)	11 (8.7)	26 (7.3)
	Grade 3	42 (7.4)	5 (5.6)	9 (7.1)	28 (7.9)
	Grade 4	171 (30.1)	19 (21.3)	33 (26.2)	119 (33.6)
	Grade 5	47 (8.3)	5 (5.6)	8 (6.3)	34 (9.6)

*CEAP*, Clinical-Etiological-Anatomical-Pathophysiological.

Data are presented as n (%), with percentages calculated within the overall cohort or the corresponding CEAP clinical-class stratum. Venography-based variables, treatment strategy, and classification based on any imaging modality included all 578 limbs (C0-C2, n = 90; C3, n = 127; C4-C6, n = 361). Ultrasound-based variables and venography-ultrasound concordance analyses included 569 limbs with usable paired imaging data (C0-C2, n = 89; C3, n = 126; C4-C6, n = 354).

For classification based on any imaging modality, the deep or superficial venous system was considered reflux-positive if reflux was identified by either venography or ultrasound. Cross-modality concordance was defined as exact agreement between venography and ultrasound study in the assigned four-category reflux phenotype. Complete cross-modality discordance was defined as isolated deep reflux on one modality and isolated superficial reflux on the other.

Venographic deep reflux was graded according to the Kistner classification, based on the distal anatomical extent of reflux. Ultrasound reflux duration was categorized using an investigator-defined scale: grade 0, no reflux; grade 1, >0 to 1 second; grade 2, >1 to 2 seconds; grade 3, >2 to 3 seconds; grade 4, >3 to 5 seconds; and grade 5, >5 seconds. This ultrasound grading scale was used only for descriptive analyses and was not considered equivalent to the venography-based Kistner classification. Percentages may not total 100% because of rounding.

**Fig 3:**
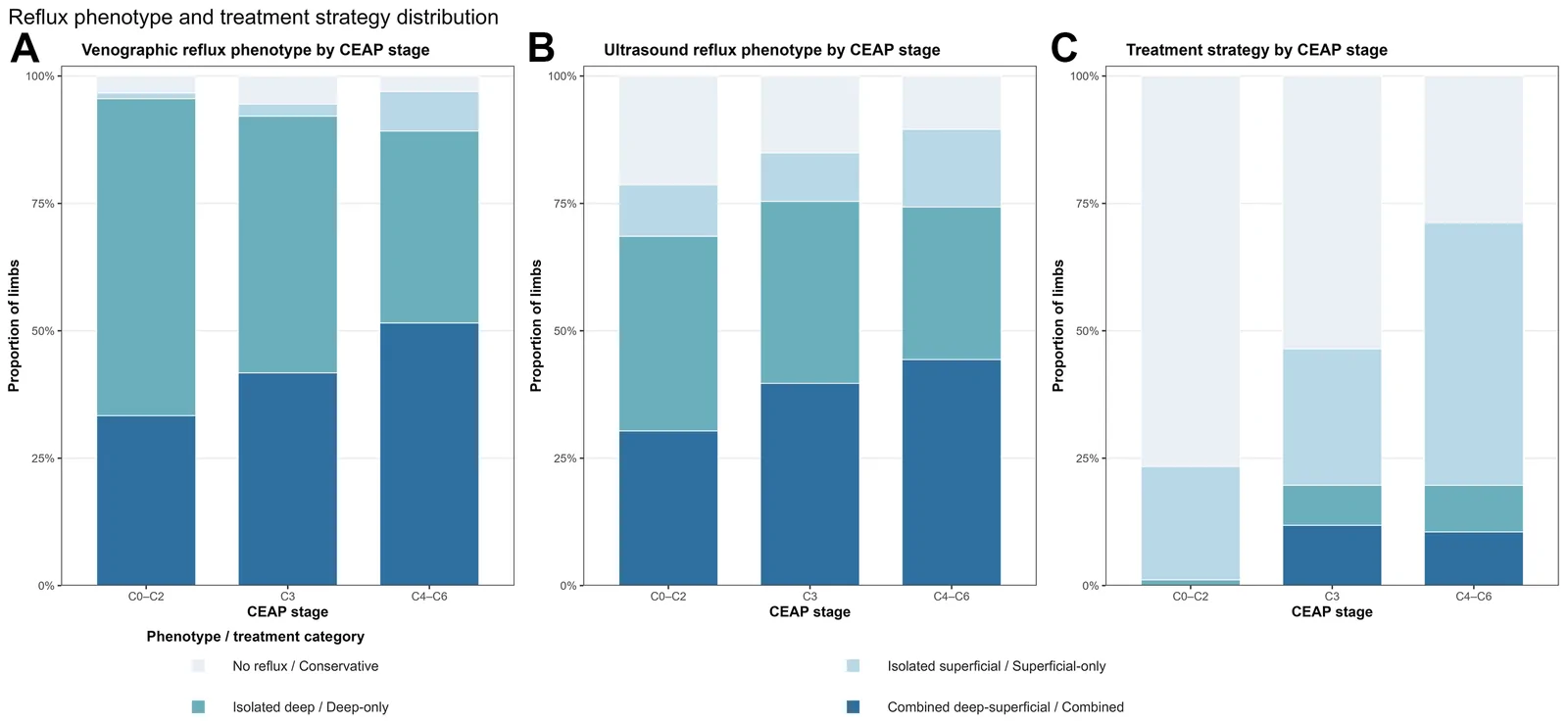
Reflux phenotype and treatment strategy distributions across CEAP clinical classes. Stacked *bar* charts show the proportional distributions of imaging-defined reflux phenotypes and treatment strategies across the C0-C2, C3, and C4-C6 CEAP clinical-class groups. **A,** Distribution of venography-defined reflux phenotypes among the 578 limbs with available venographic data. **B,** Distribution of ultrasound-defined reflux phenotypes among 569 limbs with usable ultrasound data. **C,** Distribution of treatment strategies among 578 limbs in the overall cohort. For panels **A** and **B,** reflux phenotype was classified as no definite reflux, isolated deep reflux, isolated superficial reflux, or combined deep and superficial reflux. For panel **C,** treatment strategy was classified as conservative management, deep-only intervention, superficial-only intervention, or combined deep and superficial intervention. Each *bar* represents 100% of the limbs within the corresponding *CEAP* clinical-class group. The same color sequence was used to facilitate visual comparison between imaging phenotypes and treatment categories; however, imaging-defined reflux phenotype and treatment allocation represent distinct classifications and should not be interpreted as interchangeable. *CEAP*, Clinical-Etiological-Anatomical-Pathophysiological.

### CEAP-stratified treatment effects

After adjusting for baseline VCSS, follow-up duration, center, and treatment effects showed clear variation across CEAP clinical classes (Fig 4; Table III). In C3 disease, combined intervention did not provide additional VCSS improvement compared with superficial-only intervention (adjusted β, .14; 95% CI, −1.59 to 1.87; *P* = .872). When deep-only and combined intervention were merged as any deep venous intervention, no advantage over superficial-only intervention was observed (adjusted β, −.61; 95% CI, −2.16 to 0.93; *P* = .431). By contrast, among C4-C6 limbs, combined intervention was associated with greater VCSS improvement than superficial-only intervention (adjusted β, 1.82; 95% CI, 0.49-3.14; *P* = .007). Full adjusted treatment comparisons are shown in Supplementary Table II and Supplementary Fig 2 (online only).

**Fig 4:**
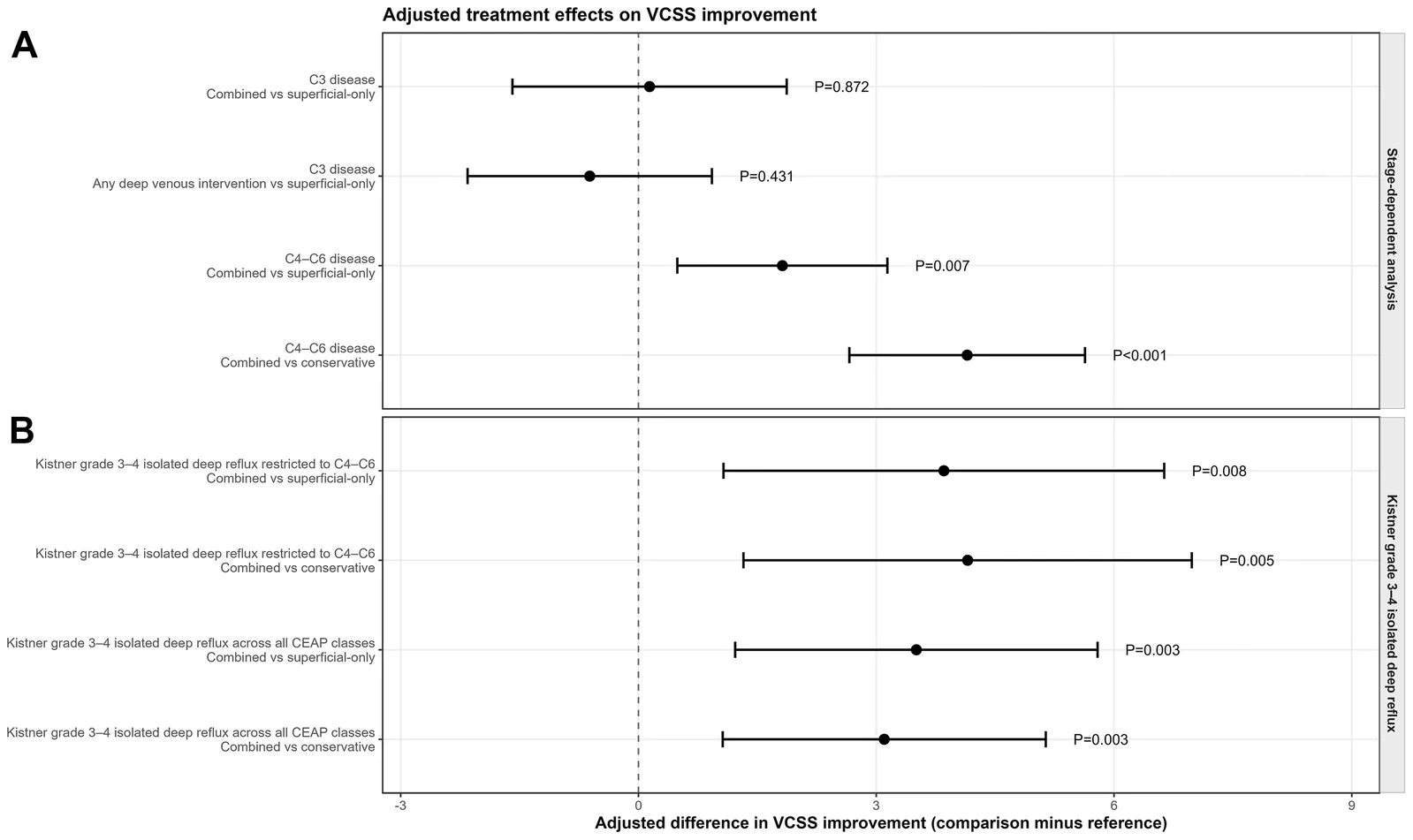
Adjusted treatment effects on VCSS improvement across CEAP and Kistner-refined populations. Forest plots display adjusted between-group differences in VCSS improvement across prespecified CEAP-stratified and Kistner-refined analysis populations. *Points* represent adjusted regression coefficients, with *horizontal lines* indicating 95% confidence intervals. The *vertical dashed line* at zero signifies no adjusted between-group difference. Positive coefficients denote greater VCSS improvement in the first-listed comparison group compared with the reference group. VCSS improvement was calculated as the baseline VCSS minus the last available follow-up VCSS. In the C3 secondary analysis, any deep venous intervention comprised deep-only intervention and combined deep and superficial intervention. The Kistner grade 3-4 populations were restricted to limbs with venography-defined isolated deep reflux. Models were adjusted for baseline VCSS, follow-up duration, and study center; the all-CEAP Kistner grade 3-4 sensitivity model was additionally adjusted for CEAP clinical class. *CEAP*, Clinical-Etiological-Anatomical-Pathophysiological; VCSS, Venous Clinical Severity Score.

**Table III:** Main adjusted treatment effects on VCSS improvement by CEAP clinical class

CEAP stratum	Model framework	Comparison	Reference	Adjusted difference in VCSS improvement (95% CI)	*P* value
C3	C3 four-group model	Combined deep and superficial treatment	Superficial-only treatment	0.14 (−1.59, 1.87)	.872
	C3 three-group model	Any deep venous intervention	Superficial-only treatment	−0.61 (−2.16, 0.93)	.431
C4-C6	C4-C6 four-group model	Combined deep + superficial treatment	Superficial-only treatment	1.82 (0.49, 3.14)	.007

*CEAP*, Clinical-Etiological-Anatomical-Pathophysiological; *CI*, confidence interval; *VCSS*, Venous Clinical Severity Score.

VCSS improvement was calculated as the baseline VCSS minus the last available follow-up VCSS; higher positive values indicate greater clinical improvement. Adjusted differences were estimated using multivariable linear regression, with positive coefficients indicating greater VCSS improvement in the comparison group than in the reference group. All models were adjusted for baseline VCSS, follow-up duration, and study center and were based on complete cases for the variables required in each model.

The C3 four-group model included conservative, deep-only, superficial-only, and combined deep and superficial treatment groups. In the C3 three-group model, the deep-only and combined treatment groups were merged as any deep venous intervention. CEAP clinical class was not included as a covariate because these analyses were conducted within CEAP clinical-class strata.

### Kistner grade 3-4 venography-defined isolated deep venous reflux

In the main C4-C6 Kistner analysis, 75 limbs exhibited venography-defined isolated deep reflux of Kistner grade 3-4; 54 of these were included in the adjusted model (Fig 4; Table IV). CEAP distributions for the C4-C6 and all-CEAP Kistner grade 3-4 populations are shown in Supplementary Tables III and IV (online only). Combined intervention was associated with greater VCSS improvement compared with superficial-only intervention (adjusted β, 3.85; 95% CI, 1.07-6.63; *P* = .008) and conservative management (adjusted β, 4.15; 95% CI, 1.32-6.98; *P* = .005). Deep-only intervention was not associated with greater VCSS improvement than superficial-only intervention.

**Table IV:** Adjusted treatment effects on VCSS improvement in Kistner grade 3-4 venography-defined isolated deep reflux

Analysis	Comparison	Reference	Adjusted difference in VCSS improvement (95% CI)	*P* value
Main analysis: C4-C6 Kistner grade 3-4 isolated deep reflux	Combined deep and superficial treatment	Superficial-only treatment	3.85 (1.07, 6.63)	.008
		Conservative treatment	4.15 (1.32, 6.98)	.005
		Deep-only treatment	1.89 (−0.84, 4.63)	.171
	Deep-only treatment	Conservative treatment	2.26 (−0.43, 4.95)	.098
	Superficial-only treatment		0.30 (−2.37, 2.97)	.822
	Deep-only treatment	Superficial-only treatment	1.96 (−0.77, 4.68)	.155
Sensitivity analysis: all CEAP Kistner grade 3-4 isolated deep reflux	Combined deep and superficial treatment	Superficial-only treatment	3.51 (1.22, 5.79)	.003
		Conservative treatment	3.10 (1.06, 5.14)	.003
		Deep-only treatment	2.08 (−0.03, 4.19)	.053
	Deep-only treatment	Conservative treatment	1.02 (−0.91, 2.95)	.296
	Superficial-only treatment		−0.41 (−2.39, 1.58)	.686
	Deep-only treatment	Superficial-only treatment	1.43 (−0.77, 3.63)	.201

*CEAP*, Clinical-Etiological-Anatomical-Pathophysiological; *CI*, confidence interval; *VCSS*, Venous Clinical Severity Score.

VCSS improvement was calculated as the baseline VCSS minus the last available follow-up VCSS; higher positive values indicate greater clinical improvement. Adjusted differences were estimated using multivariable linear regression, where positive coefficients indicate greater VCSS improvement in the comparison group than in the reference group.

The main analysis was restricted to C4-C6 limbs with Kistner grade 3-4 venography-defined isolated deep reflux. The sensitivity analysis applied the same imaging definition across all CEAP clinical classes. Kistner grade 3-4 isolated deep reflux was defined as venographic deep reflux grade 3 or 4 with venographic superficial reflux grade 0.

All four treatment groups were retained in each model, and the reported pairwise contrasts were estimated within the same fitted model. Models were adjusted for baseline VCSS, follow-up duration, and study center and were based on complete cases for the variables required in each model. The all-CEAP sensitivity model was additionally adjusted for CEAP clinical class.

In the all-CEAP sensitivity analysis, 127 limbs met the same venographic criteria, and 94 were included in the model (Supplementary Table IV, online only). Combined intervention was associated with greater VCSS improvement than superficial-only intervention (adjusted β, 3.51; 95% CI, 1.22-5.79; *P* = .003) and conservative management (adjusted β, 3.10; 95% CI, 1.06-5.14; *P* = .003). The comparison between combined and deep-only intervention did not achieve statistical significance (*P* = .053).

### Clinical deterioration and postoperative duplex findings

Among the 442 limbs with both baseline and last follow-up VCSS assessments, 86 (19.5%) had a higher VCSS at the last follow-up than at baseline, indicating clinical deterioration. The corresponding proportions were 32.8% (59/180) in the conservative group, 10.9% (20/184) in the superficial-only group, 15.2% (5/33) in the deep-only group, and 4.4% (2/45) in the combined group. Of the 78 evaluable limbs that underwent deep venous intervention, clinical deterioration occurred in seven (9.0%). Review of the available postoperative duplex ultrasound records showed that one deep-only limb and one combined-treatment limb had satisfactory valve function at approximately 1 month after surgery, followed by recurrent reflux at approximately 6 months. However, in both cases, the recurrent reflux was less severe than pre-surgery. Notably, four of the five deep-only limbs with clinical deterioration had undergone surgery approximately 8 to 10 years earlier.

## Discussion

In this two-center, real-world cohort study, the benefit of any deep venous intervention depended strongly on patient selection. C3 disease showed no additional VCSS improvement with combined or any deep venous intervention, whereas C4-C6 disease showed greater improvement with combined intervention. The largest adjusted differences were observed in limbs with Kistner grade 3-4 venography-defined isolated deep reflux. These findings support considering the CEAP clinical class in treatment selection. C3 disease represents an important boundary because edema may reflect lymphatic dysfunction, obesity, impaired calf muscle pump function, poor adherence to compression therapy, systemic factors, venous reflux, or a combination of these factors.^14^^,^^22^^,^^23^ Therefore, imaging-detected deep reflux alone may be insufficient to justify routine deep intervention in C3 disease. By contrast, C4-C6 clinical classes reflect established tissue consequences of chronic venous hypertension, where superficial-only intervention may be insufficient in selected limbs with higher-grade reflux.^24^^,^^25^

The observed pattern of discordance—most commonly, combined reflux on one imaging modality and isolated deep or superficial reflux on the other—raises the possibility that clinically relevant reflux components may be underdetected when assessment relies on a single modality. However, because neither venography nor duplex ultrasound study served as an independent reference standard, these discrepancies cannot be regarded as confirmed missed diagnoses. Rather, they highlight the need to develop and prospectively validate more sensitive and standardized imaging technologies tailored to venous hemodynamics, ideally integrating anatomical and functional information. Incorporating such technologies into venous surgical practice may improve reflux phenotyping, preoperative planning, and patient selection for deep and superficial venous interventions.

Kistner grade 3-4 reflux appeared to further refine selection beyond CEAP clinical class. The larger adjusted differences observed in venography-defined isolated deep reflux should not be interpreted as an automatic indication for combined treatment because treatment allocation was nonrandomized and may reflect clinician selection, patient preference, operative feasibility, and residual confounding. However, because the contrast between combined and deep-only intervention did not reach statistical significance, the present findings do not establish that concomitant superficial treatment is superior to deep venous reconstruction alone. The present data do not support a specific baseline VCSS threshold for common femoral vein reconstruction; treatment decisions should instead integrate CEAP clinical class, venographic reflux extent, operative feasibility, surgical risk, and patient preference. Kistner grade 3-4 should be viewed as a candidate marker for identifying patients who may benefit from combined intervention.

Clinical deterioration during follow-up was observed in a minority of limbs undergoing deep venous intervention, with a higher last follow-up VCSS than baseline in 9.0% of evaluable limbs. In the two limbs with serial postoperative duplex ultrasound records, repaired valve function was satisfactory at approximately 1 month, whereas reflux recurred by approximately 6 months, although it remained less severe than before surgery. In addition, four of the five deep-only limbs with clinical deterioration had undergone surgery approximately 8 to 10 years earlier. These findings raise the possibility that late deterioration may reflect recurrent reflux, progression of venous disease, limited long-term durability of valve reconstruction, or temporal differences in surgical technique and experience during the earlier study period. However, an increase in VCSS cannot be regarded as direct evidence of valve-repair failure because VCSS can also be influenced by superficial venous disease, compression adherence, ulcer recurrence, and other clinical factors. Moreover, the observed deterioration rates were descriptive and unadjusted, and the nonrandomized treatment allocation precludes direct comparison of long-term durability between the treatment groups. Prospective studies incorporating scheduled serial duplex ultrasound studies and repeated clinical assessments are required to clarify the relationship between repaired valve function and long-term clinical outcomes.

These results refine existing guideline positions, which generally favor superficial venous surgery first and remain cautious about deep venous valve repair.^13^^,^^14^^,^^26^^,^^27^ A potential clinical framework may consider CEAP clinical class when assessing whether deep venous intervention should be added, with venographic reflux phenotype and Kistner grade used to further inform treatment selection.

External sleeve wrapping should be used selectively rather than as a routine component of femoral vein valve repair. In our technique, the prefabricated external sleeve was not applied under tension; it was intentionally sutured with slight laxity to accommodate limited postoperative venous expansion and to reduce the risk of excessive constriction. Although the sleeve may provide structural support when the venous wall is dilated or unable to maintain adequate valve coaptation, implantation of an external prosthetic material may still cause foreign-body reactions and progressive perivenous fibrosis, potentially reducing venous compliance. These potential disadvantages should be balanced against the expected mechanical benefit, and longer-term imaging and safety data are needed to evaluate valve durability and sleeve-related complications.

A strength of this study was its venography-based cohort definition, which allowed for detailed anatomical assessment and stringent exclusion of post-thrombotic, iliac obstructive or compressive, and pelvic venous disorders. However, several limitations should be acknowledged. First, this retrospective observational study remains vulnerable to residual confounding by indication, despite stratification and multivariable adjustment. Conservative management after cohort entry followed a standardized protocol at both centers. However, the conservative treatment received by surgically treated patients before the index operation could not be fully reconstructed retrospectively. Its duration, intensity, and adherence varied, and no uniform minimum treatment period or formal treatment-failure criterion was required before surgery. Second, some key subgroups, particularly four-group comparisons within the Kistner grade 3-4 population, had limited sample sizes and wide confidence intervals. Third, treatment groups included heterogeneous deep and superficial procedures, precluding comparison of specific operative techniques. Fourth, imaging assessment may have been affected by technique, interpretation, missing data, and limited venography-ultrasound concordance. Formal intraobserver and interobserver reproducibility was not assessed for the routine duplex ultrasound examinations. Although venographic studies were independently reviewed by two blinded physicians with disagreements adjudicated by a third physician, the reviewer-specific preliminary classifications were not retained, precluding retrospective calculation of a quantitative interobserver agreement coefficient. A postoperative duplex ultrasound study was routinely obtained after deep venous reconstruction to assess venous patency and repaired valve competence. However, postoperative imaging was not uniformly available in the superficial-only group because many patients underwent follow-up at local hospitals or did not undergo repeat imaging after clinical improvement, and the timing of follow-up examinations was not standardized. Therefore, the persistence or resolution of deep and superficial reflux and direct between-group comparison of postoperative valve function could not be assessed reliably. Fifth, follow-up duration varied, and analyses used baseline and last available VCSS assessments rather than repeated longitudinal trajectories; treatment effects, therefore, represent adjusted differences in cumulative improvement rather than time-varying effects. In addition, VCSS was clinician-assessed, and no standardized patient-reported outcome measure was consistently available across the cohort; therefore, patient-perceived symptoms and health-related quality of life could not be evaluated directly. Finally, because the cohort was restricted to primary venous valvular incompetence and excluded post-thrombotic, iliocaval obstructive or compressive, pelvic venous, nutcracker, and arterial occlusive disorders, the findings may not be generalizable to other forms of CVD.

These limitations also define clear priorities for future research. We plan to conduct prospective studies using prespecified eligibility criteria, treatment protocols, outcome definitions, and statistical analysis plans, along with standardized serial clinical and duplex ultrasound follow-up. Although blinding of patients and operating surgeons is not feasible for surgical interventions, future studies should incorporate independent blinded outcome assessment, centralized blinded imaging review with formal interobserver reproducibility evaluation, and blinded statistical analysis where feasible. Particular attention should be given to scheduled duplex ultrasound study after superficial venous intervention to determine whether deep venous reflux and impaired valve coaptation resolve, improve, or persist after correction of the superficial component and to relate these imaging trajectories to VCSS, patient-reported outcomes, recurrence, and reintervention. Leveraging the clinical volume and collaborative network of our institutions, we intend to expand this work into a multicenter prospective program and ultimately a multicenter randomized controlled trial in carefully selected patients, with the goal of generating higher-level evidence to guide the treatment of primary lower-limb venous valvular incompetence.

## Conclusions

Deep venous intervention should be considered selectively in primary lower-limb venous valvular incompetence. The findings do not support routine deep venous intervention in C3 disease, whereas combined deep and superficial intervention was associated with greater benefit in C4-C6 disease. Larger adjusted differences were observed in limbs with Kistner grade 3-4 venography-defined isolated deep reflux. These findings support a CEAP clinical class-guided and Kistner grade-refined framework for selectively considering deep intervention in patients with advanced clinical burden, higher-grade reflux, and a greater likelihood of benefit.

## Author Contributions

Conception and design: BZ, YW

Analysis and interpretation: BZ

Data collection: BZ, JZhu, SW, RB, YL, HZ, JZha, LZ, ML

Writing the article: BZ

Critical revision of the article: JZhu, SW, RB, YL, HZ, JZha, LZ, ML, YW

Final approval of the article: BZ, JZhu, SW, RB, YL, HZ, JZha, LZ, ML, YW

Statistical analysis: BZ, JZhu, SW

Obtained funding: YW

Overall responsibility: YW

BZ and JZ contributed equally to this article and share co-first authorship.

## Declaration of generative AI and AI-assisted technologies in the writing process

During the preparation of this work, the authors used ChatGPT to assist with English grammar correction and text formatting. After using this tool, the authors reviewed and edited the content as needed and take full responsibility for the content of the publication.

## Disclosures

None.

## Figures and Tables

**Supplementary Fig 1:**
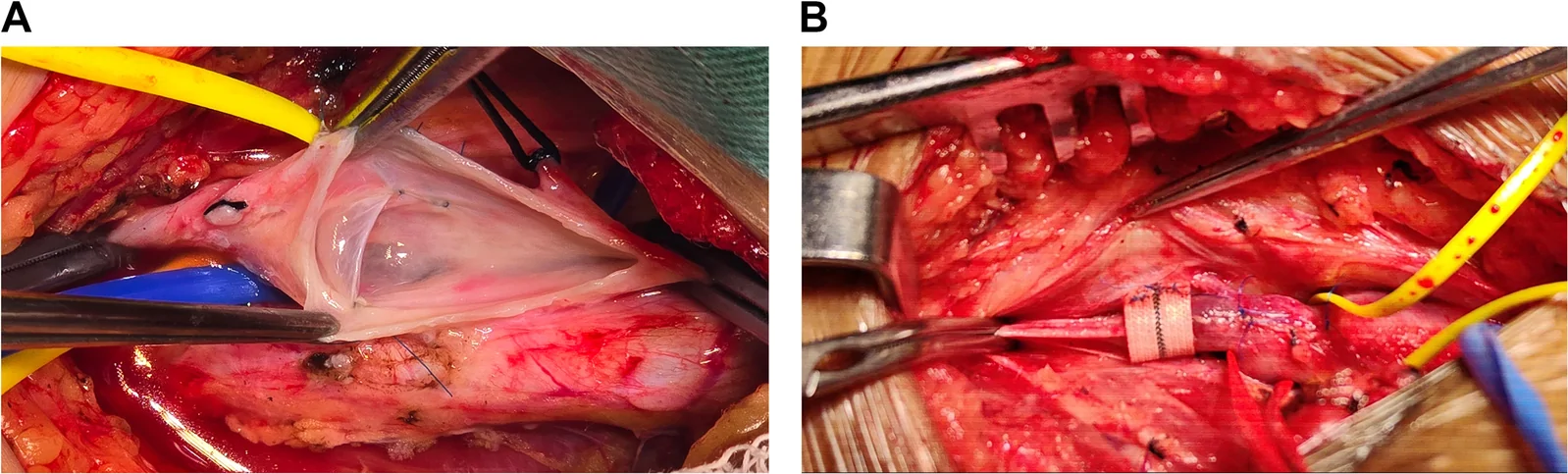


**Supplementary Fig 2:**