# Supplementary material for: CEAP-guided and Kistner-refined selection of deep venous intervention for primary lower-limb venous valvular incompetence: A two-center cohort study
Source: J Vasc Surg Venous Lymphat Disord. 2026 Aug 24;15(1):102604. doi: 10.1016/j.jvsv.2026.102604 (PMC13634078; doi:10.1016/j.jvsv.2026.102604)
Supplement: Supplementary file 1 [file mmc1.docx]

Supplementary Table S1. Detailed venographic reflux phenotype and Kistner grade distributions across key treatment cohorts.

| **Analysis cohort** | **Group** | **Venographic reflux phenotype** | **Kistner grade 0** | **Kistner grade 1** | **Kistner grade 2** | **Kistner grade 3** | **Kistner grade 4** | **Kistner grade 3–4 subtotal** | **Phenotype total** | **Treatment-group sample size, n** |
| --- | --- | --- | --- | --- | --- | --- | --- | --- | --- | --- |
| **C0–C2 conservative versus surgery cohorts** | **C0–C2 conservative** | No venographic reflux | 2 (2.9%) | - | - | - | - | - | 2 (2.9%) | 69 |
|  |  | Isolated deep reflux | - | 19 (27.5%) | 15 (21.7%) | 14 (20.3%) | - | 14 (20.3%) | 48 (69.6%) |  |
|  |  | Isolated superficial reflux | - | - | - | 1 (1.4%) | - | 1 (1.4%) | 1 (1.4%) |  |
|  |  | Combined deep and superficial reflux | - | - | 8 (11.6%) | 7 (10.1%) | 3 (4.3%) | 10 (14.5%) | 18 (26.1%) |  |
|  | **C0–C2 surgery** | No venographic reflux | 1 (4.8%) | - | - | - | - | - | 1 (4.8%) | 21 |
|  |  | Isolated deep reflux | - | 2 (9.5%) | 2 (9.5%) | 4 (19.0%) | - | 4 (19.0%) | 8 (38.1%) |  |
|  |  | Isolated superficial reflux | - | - | - | - | - | - | - |  |
|  |  | Combined deep and superficial reflux | - | 1 (4.8%) | - | 3 (14.3%) | 8 (38.1%) | 11 (52.4%) | 12 (57.1%) |  |
| **C3 direct boundary analysis** | **C3 superficial only (combined comparison)** | No venographic reflux | 2 (5.9%) | - | - | - | - | - | 2 (5.9%) | 34 |
|  |  | Isolated deep reflux | - | 1 (2.9%) | 3 (8.8%) | 1 (2.9%) | - | 1 (2.9%) | 5 (14.7%) |  |
|  |  | Isolated superficial reflux | - | - | - | 2 (5.9%) | - | 2 (5.9%) | 2 (5.9%) |  |
|  |  | Combined deep and superficial reflux | - | - | 3 (8.8%) | 18 (52.9%) | 4 (11.8%) | 22 (64.7%) | 25 (73.5%) |  |
|  | **C3 combined deep and superficial** | No venographic reflux | - | - | - | - | - | - | - | 15 |
|  |  | Isolated deep reflux | - | - | - | 4 (26.7%) | 1 (6.7%) | 5 (33.3%) | 5 (33.3%) |  |
|  |  | Isolated superficial reflux | - | - | - | - | - | - | - |  |
|  |  | Combined deep and superficial reflux | - | - | - | 7 (46.7%) | 3 (20.0%) | 10 (66.7%) | 10 (66.7%) |  |
| **C3 expanded boundary analysis** | **C3 superficial only (deep-involved comparison)** | No venographic reflux | 2 (5.9%) | - | - | - | - | - | 2 (5.9%) | 34 |
|  |  | Isolated deep reflux | - | 1 (2.9%) | 3 (8.8%) | 1 (2.9%) | - | 1 (2.9%) | 5 (14.7%) |  |
|  |  | Isolated superficial reflux | - | - | - | 2 (5.9%) | - | 2 (5.9%) | 2 (5.9%) |  |
|  |  | Combined deep and superficial reflux | - | - | 3 (8.8%) | 18 (52.9%) | 4 (11.8%) | 22 (64.7%) | 25 (73.5%) |  |
|  | **C3 deep-involved** | No venographic reflux | - | - | - | - | - | - | - | 25 |
|  |  | Isolated deep reflux | - | - | 1 (4.0%) | 11 (44.0%) | 1 (4.0%) | 12 (48.0%) | 13 (52.0%) |  |
|  |  | Isolated superficial reflux | - | - | - | - | - | - | - |  |
|  |  | Combined deep and superficial reflux | - | - | - | 8 (32.0%) | 4 (16.0%) | 12 (48.0%) | 12 (48.0%) |  |
| **C4–C6 combined versus superficial-only cohorts** | **C4–C6 combined deep and superficial** | No venographic reflux | - | - | - | - | - | - | - | 38 |
|  |  | Isolated deep reflux | - | 2 (5.3%) | 1 (2.6%) | 13 (34.2%) | 2 (5.3%) | 15 (39.5%) | 18 (47.4%) |  |
|  |  | Isolated superficial reflux | - | - | - | - | - | - | - |  |
|  |  | Combined deep and superficial reflux | - | - | - | 13 (34.2%) | 7 (18.4%) | 20 (52.6%) | 20 (52.6%) |  |
|  | **C4–C6 superficial only** | No venographic reflux | 5 (2.7%) | - | - | - | - | - | 5 (2.7%) | 186 |
|  |  | Isolated deep reflux | - | 9 (4.8%) | 8 (4.3%) | 14 (7.5%) | 1 (0.5%) | 15 (8.1%) | 32 (17.2%) |  |
|  |  | Isolated superficial reflux | - | 1 (0.5%) | 2 (1.1%) | 16 (8.6%) | 5 (2.7%) | 21 (11.3%) | 24 (12.9%) |  |
|  |  | Combined deep and superficial reflux | - | - | 4 (2.2%) | 86 (46.2%) | 35 (18.8%) | 121 (65.1%) | 125 (67.2%) |  |

**Notes:** Data are presented as n (%), with percentages calculated using the total number of limbs in each treatment group as the denominator. Each limb was assigned to one venographic reflux phenotype. For combined deep and superficial reflux, the reported grade was the higher of the deep and superficial venographic reflux grades. A dash indicates no limbs. Analyses were performed at the limb level.

**Abbreviation:** CEAP, Clinical–Etiological–Anatomical–Pathophysiological.

Supplementary Table S2. Full adjusted treatment comparisons for VCSS improvement.

| **Analysis cohort** | **Model set** | **Comparison** | **Adjusted beta** | **95% CI** | **P value** |
| --- | --- | --- | --- | --- | --- |
| **C3 disease** | **Direct boundary analysis** | Combined vs superficial-only intervention | 0.140388482857582 | -1.59 to 1.87 | 0.872 |
|  | **Expanded boundary analysis** | Any deep venous intervention vs superficial-only intervention | -0.614410195507097 | -2.16 to 0.93 | 0.431 |
| **C4–C6 disease** | **Main stage analysis** | Combined vs superficial-only intervention | 1.81571268392762 | 0.49 to 3.14 | 0.007 |
|  |  | Combined vs conservative management | 4.14739847938429 | 2.66 to 5.63 | <0.001 |
|  |  | Deep-only vs conservative management | 2.44692312653988 | 0.82 to 4.07 | 0.003 |
|  |  | Superficial-only vs conservative management | 2.33168579545667 | 1.30 to 3.36 | <0.001 |
|  |  | Combined vs deep-only intervention | 1.70047535284441 | -0.09 to 3.49 | 0.063 |
|  |  | Deep-only vs superficial-only intervention | 0.115237331083205 | -1.35 to 1.58 | 0.877 |
| **Kistner grade 3–4 isolated deep reflux restricted to C4–C6** | **Main Kistner phenotype analysis** | Combined vs superficial-only intervention | 3.8533539980018 | 1.07 to 6.63 | 0.008 |
|  |  | Combined vs conservative management | 4.15313755673382 | 1.32 to 6.98 | 0.005 |
|  |  | Deep-only vs conservative management | 2.25828443977368 | -0.43 to 4.95 | 0.098 |
|  |  | Superficial-only vs conservative management | 0.299783558732013 | -2.37 to 2.97 | 0.822 |
|  |  | Combined vs deep-only intervention | 1.89485311696014 | -0.84 to 4.63 | 0.171 |
|  |  | Deep-only vs superficial-only intervention | 1.95850088104167 | -0.77 to 4.68 | 0.155 |
| **Kistner grade 3–4 isolated deep reflux across all CEAP classes** | **All-CEAP sensitivity analysis with CEAP adjustment** | Combined vs superficial-only intervention | 3.50652684794592 | 1.22 to 5.79 | 0.003 |
|  |  | Combined vs conservative management | 3.10114906458591 | 1.06 to 5.14 | 0.003 |
|  |  | Deep-only vs conservative management | 1.02194959102415 | -0.91 to 2.95 | 0.296 |
|  |  | Superficial-only vs conservative management | -0.405377783360017 | -2.39 to 1.58 | 0.686 |
|  |  | Combined vs deep-only intervention | 2.07919947356175 | -0.03 to 4.19 | 0.053 |
|  |  | Deep-only vs superficial-only intervention | 1.42732737438417 | -0.77 to 3.63 | 0.201 |

**Notes:** VCSS improvement was calculated as baseline VCSS minus the last available follow-up VCSS; positive adjusted coefficients indicate greater improvement in the first-listed group than in the reference group. Models were adjusted for baseline VCSS, follow-up duration, and study center and were based on complete cases. The all-CEAP Kistner grade 3–4 model was additionally adjusted for CEAP clinical class. In the C3 secondary analysis, deep-only and combined intervention were merged as any deep venous intervention.

**Abbreviations:** CEAP, Clinical–Etiological–Anatomical–Pathophysiological; CI, confidence interval; VCSS, Venous Clinical Severity Score.

Supplementary Table S3. CEAP distribution among C4–C6 limbs with Kistner grade 3–4 venography-defined isolated deep reflux.

| **Treatment group** | **CEAP C4** | **CEAP C5** | **CEAP C6** | **CEAP C4–C6 subtotal** | **Total limbs, n** |
| --- | --- | --- | --- | --- | --- |
| Conservative treatment | 21 (80.8%) | 2 (7.7%) | 3 (11.5%) | 26 (100.0%) | 26 |
| Superficial-only intervention | 11 (73.3%) | 2 (13.3%) | 2 (13.3%) | 15 (100.0%) | 15 |
| Deep-only intervention | 7 (36.8%) | 1 (5.3%) | 11 (57.9%) | 19 (100.0%) | 19 |
| Combined deep and superficial intervention | 6 (40.0%) | — | 9 (60.0%) | 15 (100.0%) | 15 |
| Overall | 45 (60.0%) | 5 (6.7%) | 25 (33.3%) | 75 (100.0%) | 75 |

**Notes:** Data are presented as n (%), with percentages calculated within each treatment group. The table includes all C4–C6 limbs with Kistner grade 3–4 venography-defined isolated deep reflux, defined as deep venographic reflux grade 3 or 4 with superficial venographic reflux grade 0. A dash indicates no limbs. Analyses were performed at the limb level.

**Abbreviation:** CEAP, Clinical–Etiological–Anatomical–Pathophysiological.

Supplementary Table S4. CEAP distribution among all-CEAP limbs with Kistner grade 3–4 venography-defined isolated deep reflux.

| **Treatment group** | **CEAP C0** | **CEAP C1** | **CEAP C2** | **CEAP C3** | **CEAP C0–C3 subtotal** | **CEAP C4** | **CEAP C5** | **CEAP C6** | **CEAP C4–C6 subtotal** | **Total limbs, n** |
| --- | --- | --- | --- | --- | --- | --- | --- | --- | --- | --- |
| Conservative treatment | 6 (9.8%) | — | 8 (13.1%) | 21 (34.4%) | 35 (57.4%) | 21 (34.4%) | 2 (3.3%) | 3 (4.9%) | 26 (42.6%) | 61 |
| Superficial-only intervention | 1 (5.3%) | — | 2 (10.5%) | 1 (5.3%) | 4 (21.1%) | 11 (57.9%) | 2 (10.5%) | 2 (10.5%) | 15 (78.9%) | 19 |
| Deep-only intervention | — | — | 1 (3.7%) | 7 (25.9%) | 8 (29.6%) | 7 (25.9%) | 1 (3.7%) | 11 (40.7%) | 19 (70.4%) | 27 |
| Combined deep and superficial intervention | — | — | — | 5 (25.0%) | 5 (25.0%) | 6 (30.0%) | — | 9 (45.0%) | 15 (75.0%) | 20 |
| Overall | 7 (5.5%) | — | 11 (8.7%) | 34 (26.8%) | 52 (40.9%) | 45 (35.4%) | 5 (3.9%) | 25 (19.7%) | 75 (59.1%) | 127 |

**Notes:** Data are presented as n (%), with percentages calculated within each treatment group. The table includes limbs across all CEAP clinical classes with Kistner grade 3–4 venography-defined isolated deep reflux, defined as deep venographic reflux grade 3 or 4 with superficial venographic reflux grade 0. A dash indicates no limbs. Analyses were performed at the limb level.

**Abbreviation:** CEAP, Clinical–Etiological–Anatomical–Pathophysiological.
